# Elucidating fitness components of the invasive dermestid beetle *Trogoderma granarium* combining deterministic and stochastic demography

**DOI:** 10.1371/journal.pone.0212182

**Published:** 2019-02-14

**Authors:** Nikos E. Papanikolaou, Nickolas G. Kavallieratos, Marios Kondakis, Maria C. Boukouvala, Erifili P. Nika, Nikos Demiris

**Affiliations:** 1 Laboratory of Agricultural Zoology and Entomology, Department of Crop Science, Agricultural University of Athens, Athens, Greece; 2 Directorate of Plant Produce Protection, Greek Ministry of Rural Development and Food, Athens, Greece; 3 Benaki Phytopathological Institute, Kifissia, Athens, Greece; 4 Department of Statistics, Athens University of Economics and Business, Athens, Greece; 5 Laboratory of Organic Chemistry, Department of Chemistry, University of Ioannina, Panepistimioupolis, Ioannina, Greece; 6 Cambridge Clinical Trials Unit, School of Clinical Medicine, University of Cambridge, Coton House, Cambridge Biomedical Research Campus, Cambridge, United Kingdom; Pennsylvania State University, UNITED STATES

## Abstract

The invasive dermestid khapra beetle, *Trogoderma granarium*, is an important pest of stored products that is subject to strict phytosanitary measures. In this study, we conducted a demographic analysis of this species at 30, 35 and 40°C, combining deterministic and stochastic approaches. The net reproductive rate, the intrinsic rate of increase, the finite rate of increase and the doubling time did not differ significantly between 30 and 35°C, while at 40°C we detected negative values of the intrinsic rate of increase and the doubling time. The Briere model fit the data well with respect to the intrinsic rate of increase. Females of roughly 63, 42 and 21 days old reached their maximum reproductive potential at 30, 35 and 40°C, respectively. The stochastic models of this study allowed for checking model fit and the characterization of the most suitable distribution for each component of the process. We expect these results to have bearing on the management of *T*. *granarium* since they could be combined with models related to international trade and climatic change, alerting specialists towards early detection strategies against this species.

## Introduction

The viability of the populations of living organisms is strongly dependent on their fitness, referring to their ability to survive and reproduce in a specific environment [1]. Survival and reproduction are critical aspects of population dynamics, regulating their growth rate and allowing for several temporal fluctuations [2–4]. To this end, ecologists are often interested in understanding the patterns of these biological features in order to describe and predict populations’ performance [5–7].

Insects such as ectotherm organisms are characterised by the fact that their body temperature converges to the one of the environment they are exposed to [8]. This affects the rate of metabolism, the biochemical reactions which facilitate production and energy release, as well as the synthesis of necessary molecules that serve as structural or functional components [9,10]. In particular, temperature affects the functionality of enzymes, which in turn act as catalysts for these biochemical reactions [10]. Consequently, within a range of temperatures in which insects develop and reproduce, various biological features are affected, such as mortality, reproduction, life span, and growth rate [2,11–14]. Thus, the performance of the insects is subject to several temporal fluctuations in terms of population size through time. Understanding populations’ performance is of particular importance, as their assessment can lead to decisions on their management [15], particularly under the pressure of climatic change [16]. Hence, elucidating into fitness components can lead to a clearer understanding of an organism’s contribution to future generations and therefore its potential population development [2,6,17].

Demography represents the standard tool used for elucidating the fitness components of living organisms, as it allows for an integrated and comparative description of several biological processes, as well as an investigation on the organisms’ mortality and reproduction patterns [18]. Assuming a closed population with stable age distribution, applied demography allows for the calculation of several population parameters, tabulating the birth and death rates of the organisms of interest in a cohort life table [19]. Among these, the two most commonly used measures of fitness are the intrinsic rate of increase (*r*), which represents the rate of population increase, and the net reproductive rate (*R*_*o*_), which is typically interpreted as the average number of female offspring that a female gives birth to over her lifetime [2].

The invasive dermestid khapra beetle, *Trogoderma granarium*, is an economically important stored-product species that is subject to strict phytosanitary measures [20–24]. Native to India [25], its host range now includes Africa, Asia and Europe [26–28]. *Trogoderma granarium* is categorized as an A2 quarantine organism [24,29,30], as it is under quarantine regulation in numerous countries [29]. The rapid increase in interceptions at US ports is a cause of concern [23]. This trend is also evident in Europe considering the interceptions that have been recorded in numerous countries, mostly in central Europe, including Austria, Bulgaria, Croatia, Czech Republic, Italy, Poland, Portugal and Slovakia [29].

Despite the economic importance of *T*. *granarium*, there are no data on the demography of this species at different temperatures, which could provide valuable knowledge on its outbreaks and expansion and thus timely and effective management. In deterministic demographic models, the output of the model is fully determined by the parameter values and the initial conditions. On the other hand stochastic demographic models naturally quantify the randomness that stems from the inherent variability of the population and also allow for model assessment and exploration of the appropriate probability distribution for each element of interest. The latter serves towards our broader aim of embedding the current study within the stochastic approach to demography. This viewpoint has turned our attention to the stochastic modeling of the survival time (time until the event of interest occurs) of *T*. *granarium* and testing for statistically significant differences with respect to temperature treatments. We also extensively investigated the distribution of the time to the first birth, an event of primary interest. Our data contained a number of *T*. *granarium* beetles which gave no birth during their lifetime. Therefore, in contrast to the observations concerned with time-to-death, the time to the first birth data contained censored observations, necessitating a survival type of analysis. In addition, measuring the number of offspring of *T*. *granarium* beetles on a daily basis results in the observation of an excessive number of zeros (see the results for the details). The zero-inflated-Poisson model was fitted using a Bayesian approach, leading to accurate point and interval estimates, even in the presence of a large percentage of zeros in the sample [31].

The aim of this study is to combine the output of deterministic and stochastic demography in order to explore the survival or extinction and the patterns of mortality and reproduction of *T*. *granarium*, as well as to provide a comparative calculation of its demographic parameters in a different temperature range. Therefore, we shall test how the fitness components of *T*. *granarium* fluctuate with temperature. To this end, we propose stochastic models which have been underutilized in demographic studies, as they could provide important information on the functionality of *T*. *granarium*.

## Materials and methods

### Insects and commodity

*Trogoderma granarium* were reared on wheat at 30°C, 65% relative humidity at continuous darkness. The insect colony was established in 2014 from insects collected in Greek storage facilities [central (Thessaly) and southern (Attica)] and since then it has been kept at the Laboratory of Agricultural Zoology and Entomology of the Agricultural University of Athens. In all experiments we used pesticide-free wheat (*Triticum durum*, var. Claudio) in order to maintain insect colonies. The moisture content of wheat was 12.1%, as determined by a calibrated moisture meter (mini GAC plus, Dickey-John Europe S.A.S., Colombes, France) at the beginning of the tests.

### Experimental set-up

Samples of 1 g of cracked wheat were separately put inside each petri dish (8 cm diameter, 1.5 cm height). Wheat was cracked in a hand-mill. Two testing sieves were used to make particles of cracked wheat approximately consistent. First, the cracked wheat was sieved with a No 30 (2.36 mm openings) US standard testing sieve (Advantech Manufacturing, Inc., New Berlin, WI). Subsequently, the sifted material was sieved again with a No 10 (2.00 mm openings) US standard testing sieve (Retsch GmbH, Haan, Germany). Then, the content of the latter was used for experimentation. The quantities of 1 g were weighed with a Precisa XB3200D compact balance (Alpha Analytical Instruments, Gerakas, Greece). The closures of the dishes bore a 1.50 cm diameter circular opening in the middle that was covered by muslin gauze to allow the sufficient aeration inside the dish. The upper inner walls of the dishes were covered by polytetrafluoroethylen (60 wt % dispersion in water) (Sigma-Aldrich Chemie GmbH, Taufkirchen, Germany) to prevent the escape of larvae and adults.

To obtain eggs of *T*. *granarium*, 50 unsexed adult individuals, approximately 7 d old, were transferred from the culture to a 250 ml glass jar that contained 125 ml white soft wheat flour for 1 day. Then, the adults and eggs were separated from the flour with a No 20 and a No 60 U.S. standard testing sieves (Advantech Manufacturing, Inc., New Berlin, WI). The eggs that were remained on the mesh openings of the sieve were put in a petri dish and inspected daily at 57x total magnification of an Olympus stereomicroscope (SZX9, Bacacos S.A., Athens, Greece). Totally, 40, 48 and 433 eggs were used to obtain egg to adult development and mortality at 30, 35 and 40°C, respectively. We used higher number of eggs at 40°C due to detrimental impact of this temperature to *T*. *granarium* survival. Newly hatched *T*. *granarium* larvae were very carefully separately placed inside each dish, with a fine brush (Cotman 111 No 000, Winsor and Newton, London, UK), that contained the cracked wheat. The dishes were placed in incubators set at the respective temperature and 65% relative humidity during the entire experimental period. The duration and survival of egg, larval, and pupal stages were recorded every 24 hours. In addition, female longevity and fecundity were examined daily. Formed pairs were kept separately in petri dishes. We used 25, 26 and 36 pairs at 30, 35 and 40°C, respectively. The insects’ thermal window, i.e. the range in temperature between the minimum and maximum rate of development for individual species, is about 20°C [32]. Considering also that below 30°C larvae of *T*. *granarium* fall to diapause prolonging their life up to 8 years [26,27], and that *T*. *granariun* prefers environments with elevated temperatures [20,33], we selected 30, 35 and 40°C as the most suitable temperature range for our study. It should be noted that during the summer, air temperature in Greece may reach or potentially exceed 40°C.

### Demographic parameters

The following parameters were estimated at 30, 35 and 40°C, 65% relative humidity and continuous darkness [14,19,34,35]:

- the cohort survival to age *x*: (*l*_*x*_);

which represents the proportion of the cohort surviving from birth to exact age x.

- the age specific mortality: $q_{x}=1-\frac{l_{x+1}}{l_{x}}$;

which represents the probability of dying over period (*x*,*x*+1).

- the age specific fecundity (*m*_*x*_) by multiplying the mean number of eggs by the ratio ♀/(♀+♂) (observed by sorting 100 offspring);

which represents the averaged number of offspring produced by females at age x.

- the net reproductive rate: *R*_0_ = ∑(*l*_*x*_×*m*_*x*_);

which represents the per capita rate of offspring production in a period of time equal to cohort study period.

- the intrinsic rate of increase, the solution of: $\sum \left(e^{r_{m}\times x}\times l_{x}\times m_{x}\right)=1$;

which represents the rate of natural increase in a closed population (that has been subject to constant age-specific schedules of fertility and mortality for a long period) and has converged to be a stable population (that crude growth rate does not change over a long time).

- the finite rate of increase: $\lambda =e^{r_{m}}$;

which represents the rate at which the population will increase in each time step.

- the mean generation time: $T=\frac{\ln\;R_{0}}{r_{m}}$;

which represents the time required for the population to increase by a factor equal to the net reproductive rate.

- the doubling time: $DT=\frac{\ln\;2}{r_{m}}$;

which represents the time required for the population to double.

- the reproductive value of the females: $V_{x}=\frac{\sum_{t\geq x}(e^{-r_{m}\times t}\times l_{t}\times m_{t})}{l_{x}\times e^{-r_{m}\times x}}$;

which represents the average future production of eggs per female at age x.

- the expected remaining life time of the females $E_{x}=\frac{\sum_{t\geq x}\frac{l_{t}+l_{t+1}}{2}}{l_{x}}$.

which represents the expected remaining life time of insect at age x.

Significant differences between life table parameters at each of the examined temperature were tested via a Wald test, essentially the superposition of 95% confidence intervals (CIs). This is a general method for hypothesis testing and avoids the recent controversy with the use of p-values in the statistical literature. The CIs were obtained by bootstrapping in R [36], sampling with replacement 1000 datasets in each temperature group and re-estimating the parameters for each set. This technique avoids unnecessary asymptotic normality assumptions and estimates the CIs using the empirical 2.5% and 97.5% percentiles, yielding general and robust procedures for statistical estimation and hypothesis testing.

### Modeling temperature-dependent intrinsic rate of increase

The relationship between temperature and the intrinsic rate of increase was described by the Briere model [37], which is of the form:

$$r(T)=a\times T\times \left(T-T_{0}\right)\times {\left(T_{L}-T\right)}^{1/2},$$

where *T* denotes the ambient temperature; *α* is an estimated parameter; *T*_*0*_ is the lower and *T*_*L*_ the higher temperature in which the intrinsic rate of increase is equal to zero. We proceeded by assuming that at the temperature of 17.20°C the intrinsic rate of increase is equal to zero since no development has been detected [38] in this temperature.

The limited capacity of deterministic models in predictions concerned with alternative environmental conditions or the sensitivity of the beetles’ biochemical reactions to other environmental conditions [39] like pressure and substances of air breathing, suggest that the randomness of the mechanism based on which the *T*. *granarium* female beetles incubate can be appropriately modeled by a stochastic process as opposed to a deterministic model and this is described in the following subsection.

### Stochastic models

Survival analysis techniques were utilized in order to examine (in a life cycle generation) both (i) the time (in days) until the event of “death” and (ii) the time (in days) until the event of *T*. *granarium* females become active and lay their first egg. In both events of interest, the time until the event occurs can be considered as a non-negatively-distributed random variable [40]. In order to compare the survival times of *T*. *granarium*, kept at different incubators the predictor variable was the temperature level at 30, 35 and 40°C respectively. The survival probabilities of *T*. *granarium* for each temperature group were estimated using the Kaplan-Meier product-limit estimator [41].

In addition, some commonly used distributions in survival analysis were considered to fit a parametric survival regression model to the *T*. *granarium* data. Specifically, the distributions used were: (i) the Exponential, (ii) the Weibull, (iii) the Lognormal and (iv) the Log-logistic. The explanatory variable used in all the parametric models was the temperature. Selection of the most suitable model was based on the Akaike Information Criterion [42]. The Akaike Information Criterion (AIC) is a composite measure accounting for the goodness of fit of each model to the observations via the deviance, penalised for the model’s complexity by adding twice the number of estimated parameters. Details of the above fitted models and the Akaike Information Criterion are presented in the S1 File.

The statistical analysis of the number of eggs of *T*. *granarium* beetles was based upon the zero-inflated class of models. Herein, the underlying distribution considered for egg counts was the Poisson distribution leading to the ZIP (zero-Inflated-Poisson) model [31]. We used Bayesian methods for estimating the model parameters through the WinBUGS package [43], a general purpose software designed to run Markov Chain Monte Carlo (MCMC) simulations for a wide range of Bayesian models. The output of the ZIP model was obtained by running the WinBUGS software for 7000 samples within each temperature group, having 4000 iterations as burn-in. Specifically, burn-in refers to the initial, potentially non-stationary, portion of the Markov Chain measured in number of iterations. It pertains to the practice of discarding a number of samples at the start of the MCMC algorithm in order to allow the Markov Chain to reach its equilibrium (stationary) density which corresponds to the posterior distribution of interest.

The Zip model used is a mixture model for each of the datasets corresponding to the three different temperature levels of 30, 35 and 40^ο^C respectively. Let us denote with y_ij_ the response variable, the number of eggs laid by the i-th *T*. *granarium* beetle in the j-th day, so that j corresponds to the lifetime of the i-th beetle from its entry in the study until its death. Then, the statistical model we used posits that y_ij_ is either zero, with probability p, in which case no eggs are laid from i-th beetle in j-th day, or follows a Poisson distribution with parameter λ whence the i-th beetle generates an average of λ eggs in j-th day. The model is completed with vague priors, namely a Uniform density on the (0, 1) interval for p and a Gaussian with mean zero and large variance on log(λ).

## Results

### Demographic parameters

**Fig 1:**
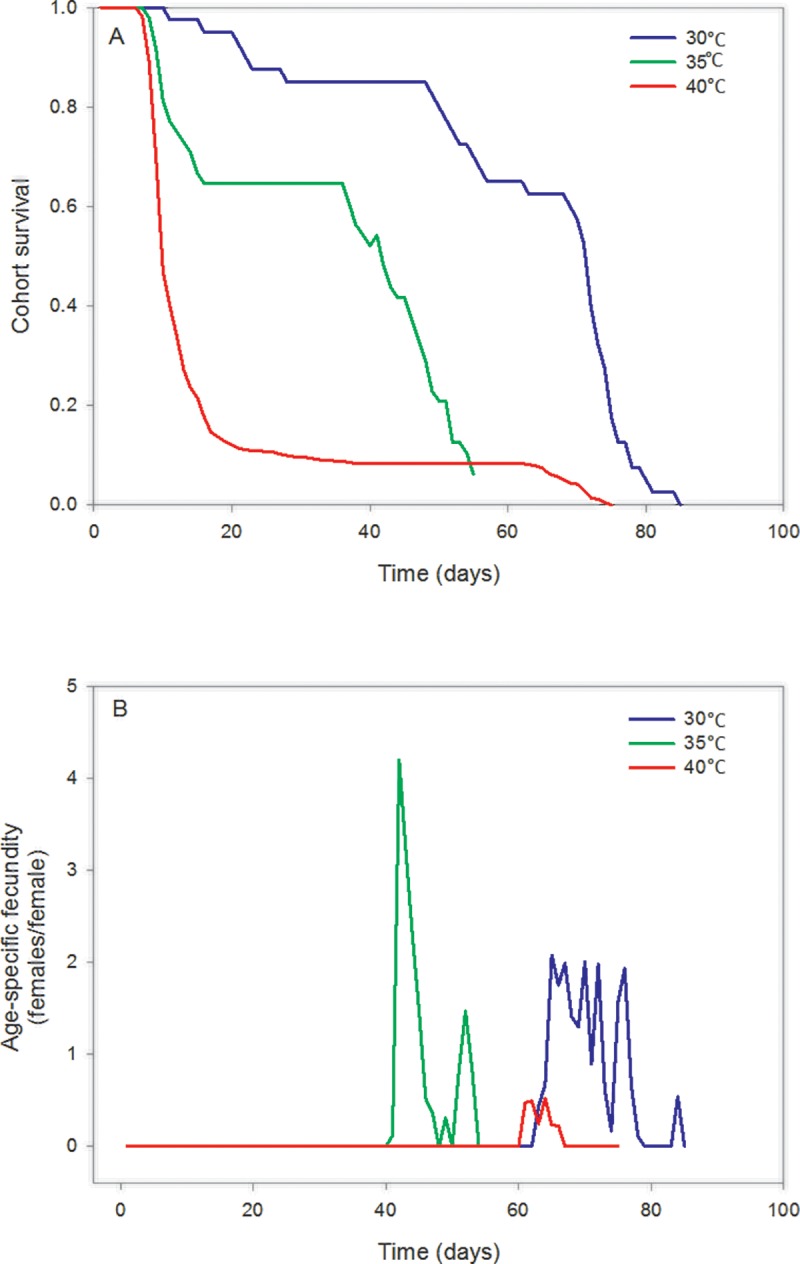
Survival and age-specific fecundity. Plot of the cohort survival (A) and the age-specific fecundity (B) of *T*. *granarium* at constant temperatures.

**Fig 2:**
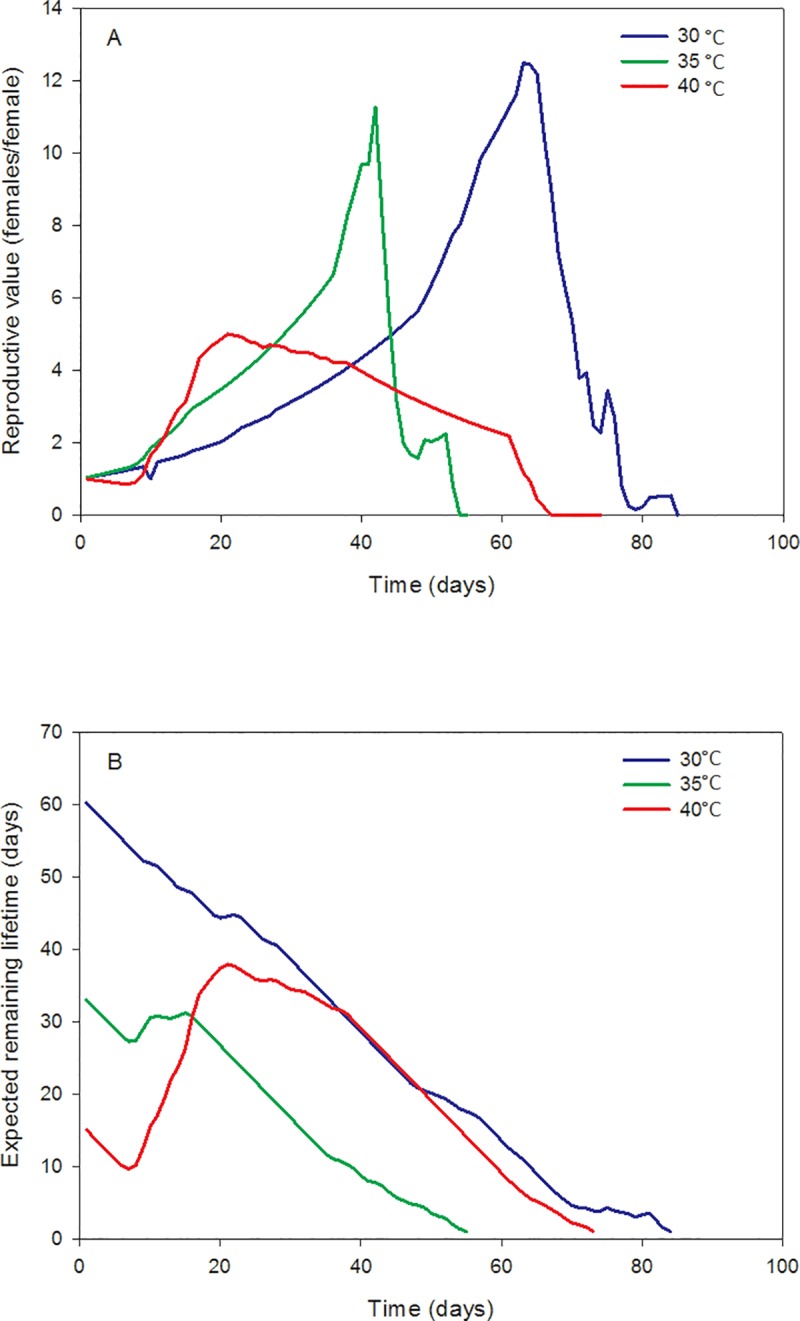
Reproductive value and expected remaining lifetime. Plot of the reproductive value (A) and the expected remaining lifetime (B) of *T*. *granarium* females at constant temperatures.

The estimated demographic parameters showed considerable variation across the different temperature regimes used in this study. This was evident based upon the inspection of the 95% confidence intervals (Table 1) which were also used for hypothesis testing. Thus, we test for statistical significance using a 5% significance level. The net reproductive rate did not differ significantly at 30 and 35°C, but was substantially lower at 40°C. The same trend was also established for the values of the intrinsic and the finite rate of increase as temperature increased from 30 and 35°C. In contrast, the corresponding values at 40°C where close to zero. The doubling time also did not differ significantly at 30 and 35°C while a significantly lower doubling time was estimated at 40°C. The mean generation time differed in all three temperatures, being significantly longer at 30°C, shorter at 35°C and an intermediate estimate at 40°C. The cohort survival decreased through time as presented in Fig 1A, while the age-specific fecundity increased until a particular age-dependent temperature, where a subsequent decrease follows (Fig 1B). In addition, females of approximately 63, 42 and 21 day-old reach their maximum reproductive potential at 30, 35 and 40°C, respectively (Fig 2A). The expected remaining life time of *T*. *granarium* females at 30, 35 and 40°C is depicted in Fig 2B, reflecting that the initial decrease in this parameter is followed by an increase -although marginally at 30°C- and an ultimately decrease. The p-values for testing the hypotheses that the demographic parameters are zero were smaller than 0.01.

**Table 1:** Demographic parameter results.

Temperature	Net reproductive rate (females/female) *R*_0_ = ∑(*l*_*x*_×*m*_*x*_)		Intrinsic rate of increase (females/female/d) $\sum \left(e^{r_{m}\times x}\times l_{x}\times m_{x}\right)=1$		Finite rate of increase $\lambda =e^{r_{m}}$		Mean generation time (d) $T=\frac{\ln\;R_{0}}{r_{m}}$		Doubling time (d) $DT=\frac{\ln\;2}{r_{m}}$	
	Mean	95% C.I.	Mean	95% C.I.	Mean	95% C.I.	mean	95% C.I.	Mean	95% C.I.
30°C	9.73	4.09–16.28	0.03	0.02–0.04	1.03	1.02–1.04	69.04	66.02–71.65	22.20	17.09–34.37
35°C	6.55	2.36–11.31	0.04	0.02–0.06	1.04	1.02–1.06	42.96	41.22–44.65	17.73	12.26–33.89
40°C	0.17	0.07–0.30	-0.03	-0.05 –-0.02	0.97	0.96–0.98	58.12	55.86–61.48	-23.08	-33.05 –-15.42

Values of net reproductive rate (*R*_*0*_), intrinsic rate of increase (*r*_*m*_), finite rate of increase (*λ*), mean generation time (*T*) and doubling time (*DT*) of *T*. *granarium* reared on wheat (mean, 95% Confidence Intervals) at constant temperatures.

**Fig 3:**
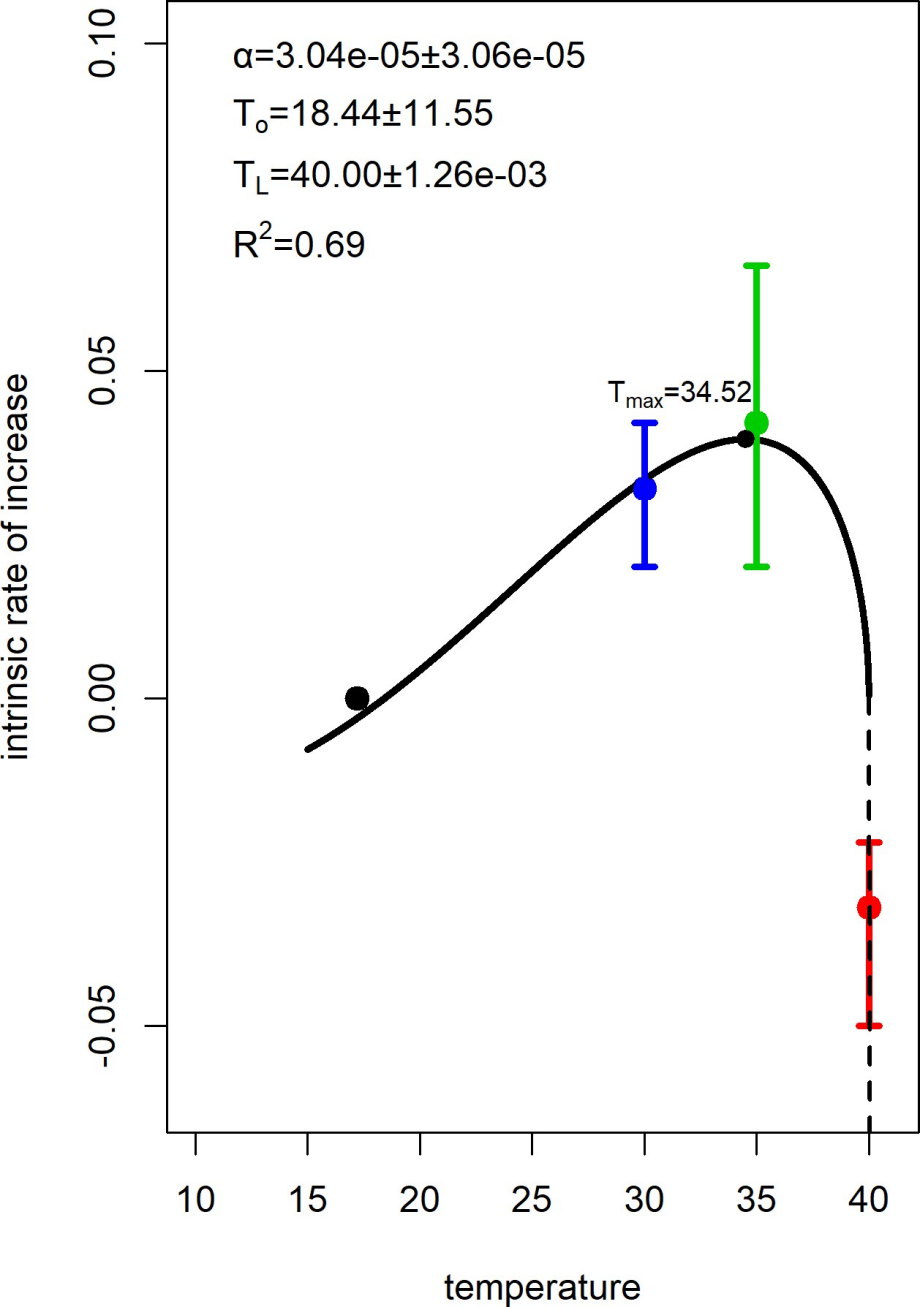
Intrinsic rate model fit. Estimated parameters and fitting of the Briere model to the intrinsic rate of increase data of *T*. *granarium*. Pointwise 95% C.I. are also depicted for each mean at 30°C, 35°C and 40°C respectively.

The Breire model fitted reasonably well (*R*^*2*^ is equal to 0.69 and standard error of the regression equal to 0.03, Fig 3) to the intrinsic rate of increase data of *T*. *granarium*. The estimated minimum and maximum temperatures where the intrinsic rate of increase is expected to reach zero are 18.44 and 40.00°C respectively, obtaining its maximum value at 34.52°C.

### Survival analysis

**Fig 4:**
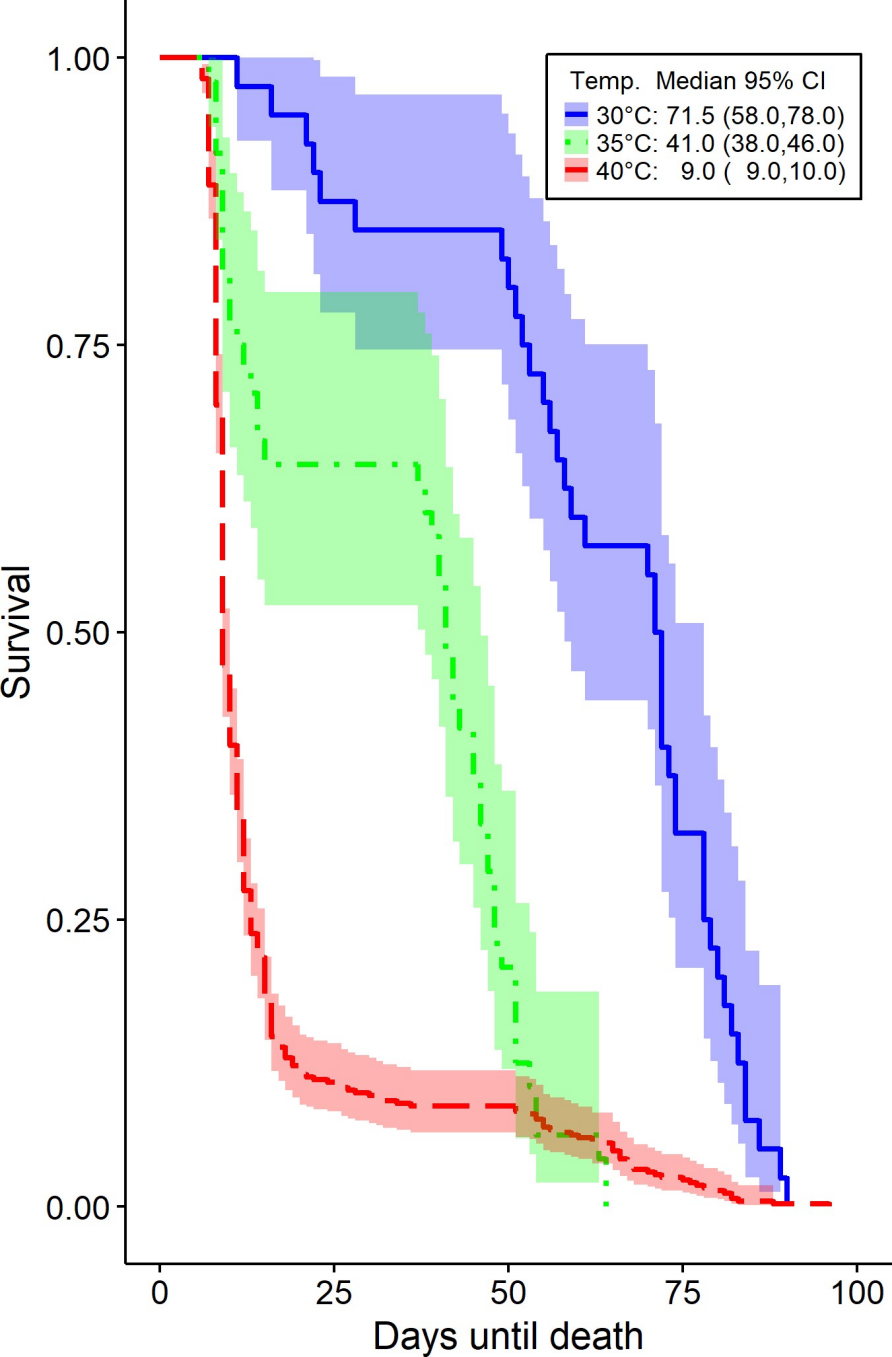
*T*. *granarium* survival. Kaplan-Meier survival curve of *T*. *granarium*time until death along with 95% confidence bands at 30°C, 35°C and 40°C respectively. No censored observations exist due to the experimental design. In the legend we report the median and its 95% C.I.

**Fig 5:**
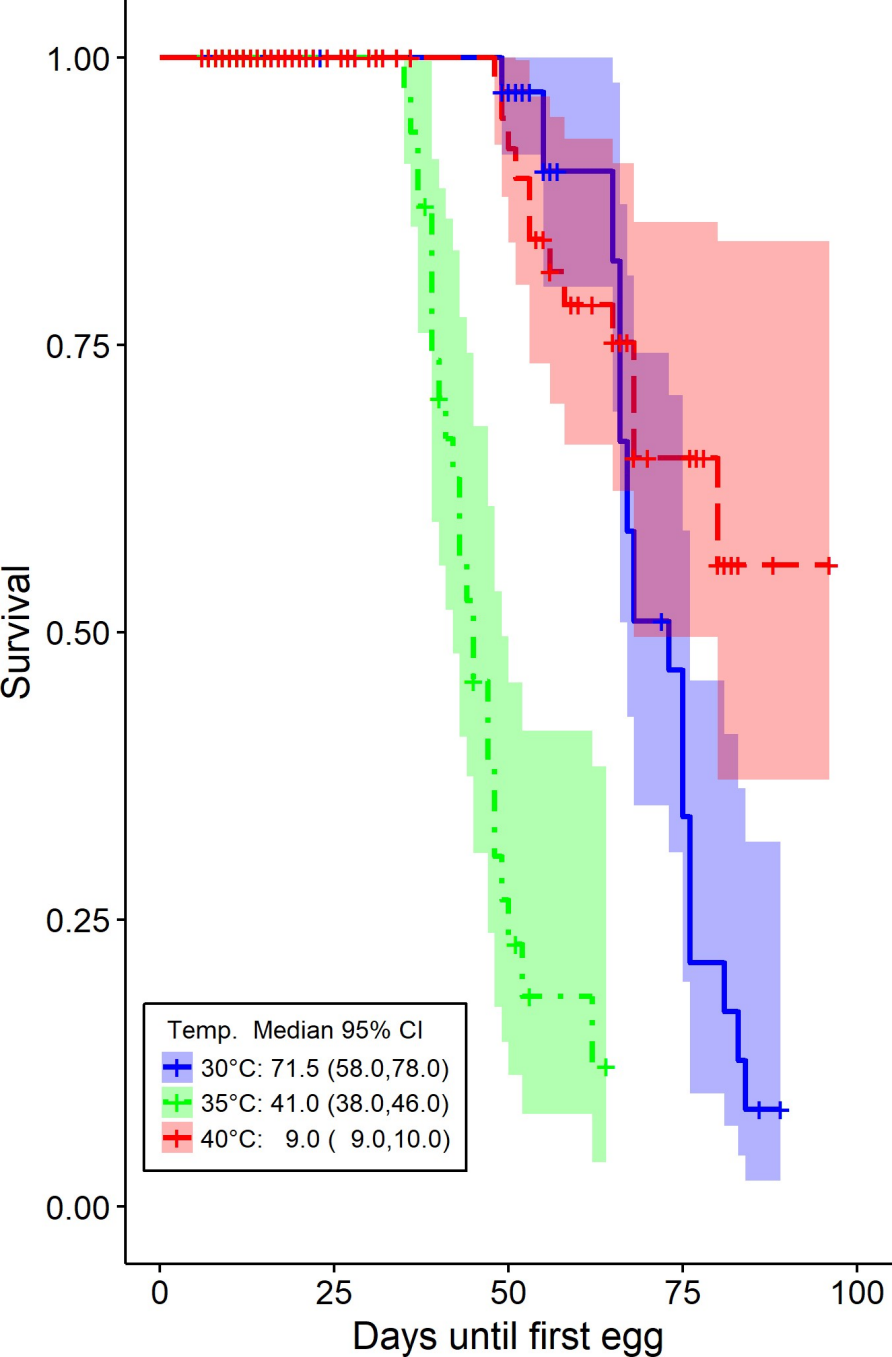
Time until *T*. *granarium* lay the first egg. Kaplan-Meier estimator of time until*T*. *granarium* lay the first egg along with their 95% confidence bands at 30°C, 35°C and 40°C respectively. Censored observations appear when *T*. *granarium* die before lay any egg and are symbolized by the “+” symbol. In the legend we report the median and its 95% C.I.

We modeled the times (in days) to two distinct types of event, the time until death, in which case there is no censoring due to the experimental design and the time until laying the first egg. The latter is subject to right censoring since some beetles die before they ever lay any egg. Hence, the median and other functionals of the survival times are affected due to censoring which is substantial for the beetles studied at 40°C. The survival times and their 95% confidence intervals are derived using the Kaplan-Meier estimators for the different temperature levels at 30, 35 and 40°C. The results are depicted on Figs 4 and 5 respectively. The means of the survival time until death of *T*. *granarium* are 62.88, 34.25 and 15.61 days while their medians diminish rapidly (Fig 4). Furthermore, the means of the time until first egg release are 71.70, 46.90 and 81.30 days while the medians decrease when the temperature rises from 30 to 35°C (Fig 5). At 40°C it is apparent (Fig 5) that the probability of *T*. *granarium* laying the first egg does not cross the 0.5 line and therefore the median time to first birth cannot be estimated.

### Parametric models

**Fig 6:**
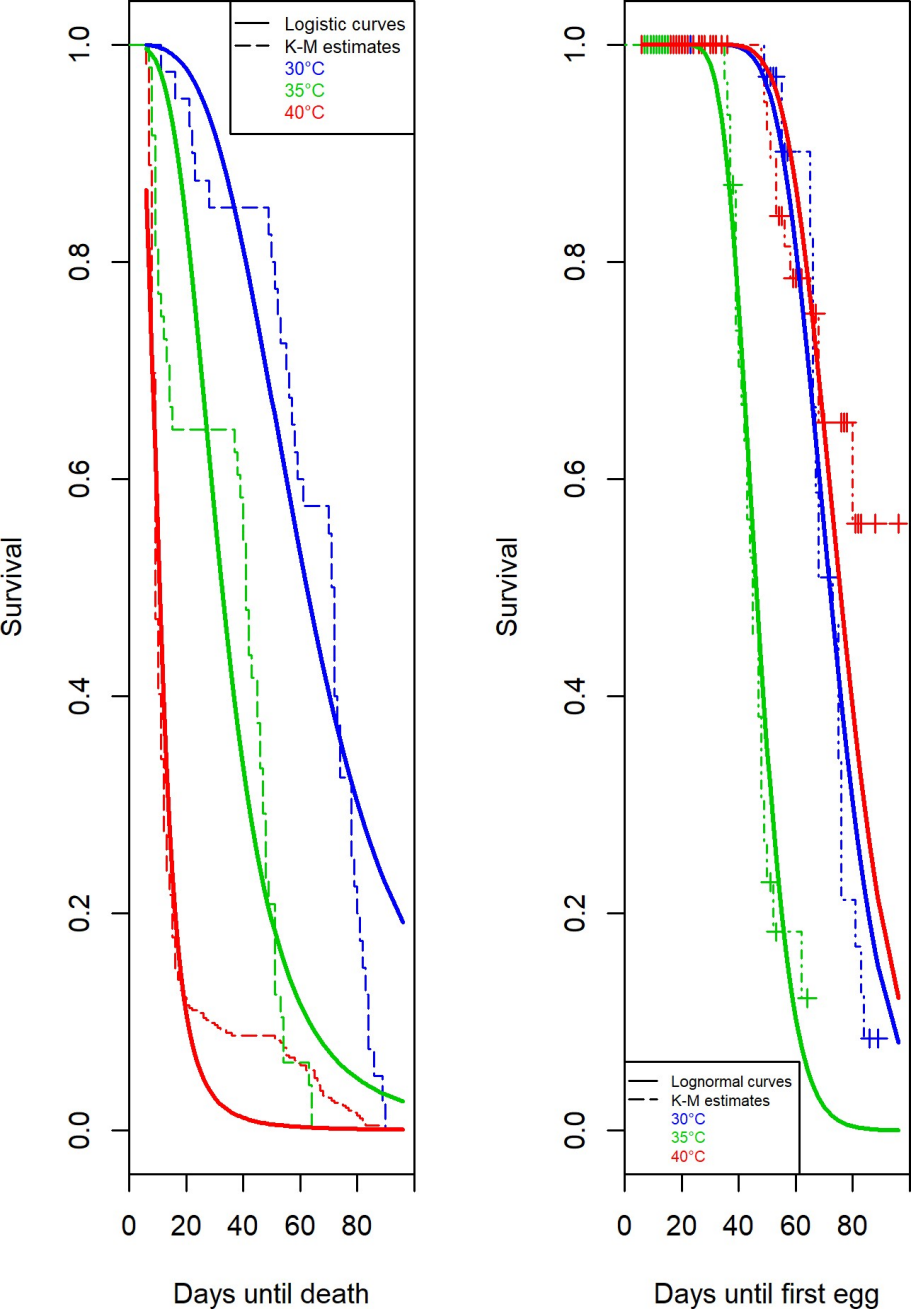
Logistic and Lognormal survival models. Logistic Model and Lognormal Model probability (Y-axis) vs. survival times of *T*. *granarium*(X-axis) until death (left) and until laying the first egg (right) along with the Kaplan-Meier survival time estimates at 30°C, 35°C and 40°C respectively.

In order to assess a parametric fit to the *T*. *granarium* survival times, the Exponential, Weibull, Lognormal and Loglogistic distributions were considered. The Akaike Information Criterion was estimated at 4096.40, 4040.70, 3772.10 and 3691.50 for the Exponential, Weibull, Lognormal and Loglogistic distributions respectively when time until death is consideredand 708.90, 20926.90, 570.10, 574.40 respectively in the case that time until first egg is studied (Table 2). It is evident that the smallest values are achieved by the Loglogistic model when the event is concerned with a *T*. *granarium* death, while the Lognormal model has the best fit when examining the time until a *T*. *granarium* lays the first egg (Fig 6).

**Table 2:** Parametric survival models.

	Model	Deviance	AIC	Number of parameters
*Survival times until death	Exponential	-2046.2	4096.4	2
	Weibull	-2017.3	4040.7	3
	Lognormal	-1883.1	3772.1	3
	Loglogistic	-1842.8	3691.5	3
**Survival times until lay first egg	Exponential	-352.4	708.9	2
	Weibull	-10460.4	20926.9	3
	Lognormal	-282.0	570.1	3
	Loglogistic	-282.4	574.4	3

Parametric survival times until death and until lay first egg (deviance, Akaike Information Criterion and degrees of freedom) for the Exponential, Weibull, Lognormal and Loglogistic models.

*Time of *T*. *granarium* until death are considered.

**Time of *T*. *granarium* until lay first egg are considered.

### Bayesian analysis of the number of eggs

**Fig 7:**
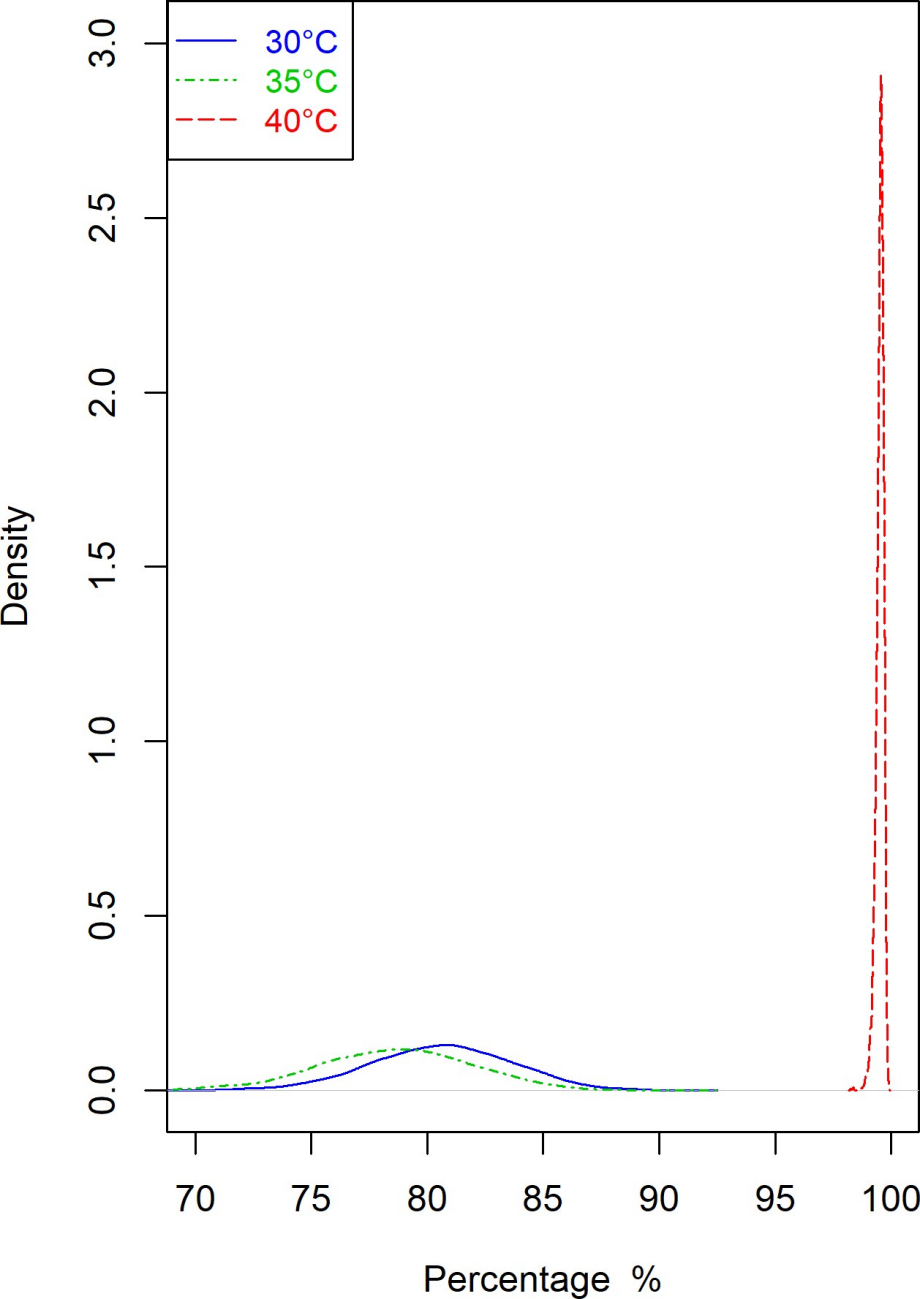
Probability of excess zeros. Plot of the posterior densities (Y-axis) for the probability of excess zeros vs. the observed percentage of zeros (X-axis), at 30°C, 35°C and 40°C respectively.

The percentage of zeros in the number of *T*. *granarium* offspring for all temperature groups is over 0.90 (Table 3). As depicted on Fig 7, the probability that *T*. *granarium* beetle lays no egg is 0.81 (with a 95% credible interval (CrI) of 0.74–0.86) at 30^ο^C, 0.79 (95% CrI: 0.72–0.85) at 35^ο^C and substantially higher at 0.997 (95% CrI: 0.991–0.998) at 40^ο^C.

**Table 3:** Probability of excess zeros.

Temperature	Pr (excess zeros)	95% Credible Intervals
30°C	0.805	0.740–0.863
35°C	0.786	0.716–0.851
40°C	0.996	0.991–0.998

Estimated probability of excess zeros and its 95% Credible Intervalin *T*. *granarium* data for each of the three temperature levels.

The posterior means (95%CrIs) for the Bernoulli parameter p are: 0.42 (0.28–0.58), 0.50 (0.35–0.64) and 0.96 (0.94–0.98) at 30^ο^C, 35^ο^C and 40^ο^C respectively.

The rate that *T*. *granarium* lay eggs in daily basis when they are active to reproduce is expressed by the lambda parameter of the Poisson distribution. Its posterior mean and 95% CrI is 0.43 and 0.40–0.46 at 30°C, 0.57 and 0.53–0.61 at 35°C, 0.14 and 0.11–0.16 at 40°C respectively. After performing the Wald test in the lambda parameters across the three temperature groups, we get “Bayesian p-values” which are less than 0.01, suggesting that there are significant differences in the number of eggs produced when comparing the three temperature groups, with the best performance observed at 35°C and the worst at 40°C respectively. Inspecting the standardised residuals suggests that no apparent pattern is emerging and no influential individual values stand out, indicating that the Zero Inflated Poisson Model has good fit and explains reasonably well the randomness that stems from the inherent variability of *T*. *granarium* data. In particular, the standardised residuals fluctuate within the expected intervals for all the temperatures. These results suggest that the differences between the observations and the fitted values of the ZIP model may be due to chance alone, leading to robust conclusions.

## Discussion

Our study revealed a number of findings on the biology of *T*. *granarium* and a comprehensive description of the survival and reproductive schedules of this invasive species in three distinct temperatures. We obtain further evidence on its growth rate, allowing for potential application in pest management. It was found that temperature seriously affects its population increase. The knowledge of the insect’s potential growth rate also facilitates for estimation of its population through time, and therefore its potential outbreak. At 40°C the value of the intrinsic rate of increase is negative, indicating that at this temperature the population tends to extinction, although *T*. *granariun* is considered a highly heat-tolerant species [44,45]. At 30 and 35°C the positive values of the intrinsic rate of increase indicates that in this temperature range *T*. *granariun* is able to increase its population size, as well as its potential to spread, becoming more harmful in stored-products.

The fact that there is a significant difference in the mean generation time between 30 and 35°C but not in the other demographic parameters may appear somewhat unexpected. The mean generation time represents the average time for a population to increase by a factor equal to the net reproductive rate. This result is biologically interpretable, since the net reproductive rate depends on cohort survival, which is lower for *T*. *granarium* at 35°C. However, as the values of the intrinsic rate of increase and the doubling time did not differ significantly, we expect that the same applies for the insect’s growth rate between these temperatures.

According to the fit of the Briere model, the minimum and maximum temperatures for *T*. *granarium* population increase are roughly 18.44 and 40.00°C respectively. In this range of temperatures *T*. *granarium* is able to increase its population. This is important for the management of this species, considering its economic importance and further spreading in the world, as well as its mass-rearing, allowing efficient breeding in the insectary [46]. The *T*. *granarium* intrinsic rate of increase shows an increasing trend until 34.52°C, where it reaches its maximum value. The subsequent decrease at higher temperatures is probably due to the determinental effect of these temperatures on its survival and reproductive capacity. According to the model’s predictions, temperatures around 34°C are optimal for population growth of *T*. *granarium*, whereas temperatures in the area of 40°C lead to population decrease. These results clearly indicate that the population development of *T*. *granarium* is strongly affected by temperature. It should be noted that elevated temperature levels, which favor the population increase of *T*. *granarium*, are responsible for potential outbreaks of this species, an issue that leads to considerable losses of the infested commodities [47]. Even when the initial population of *T*. *granarium* consists of a small number of larvae, it can increase fast under favorable temperature conditions and commodities [47]. Temperatures from 30°C to 35°C support the development of high numbers of *T*. *granarium* larvae, that is the most difficult life stage of this species to be controlled on stored commodities, especially on wheat [47,48]. Given that the efficacy of several insecticidal active ingredients against stored-product insect pest species varies among different levels of temperature [49–51], potential optimization of chemical control measures should seriously take into account the combination of toxicants and temperature when applied against *T*. *granarium*. It is recommended to control this species when its numbers are still low as a way to moderate its population growth [47]. Also, based on our findings, since the population of *T*. *granarium* decreases at 40°C, we could suggest a further rapid decline when insecticidal applications are targeted at the above temperature level on stored wheat. This is a realistic scenario, given that *T*. *granarium* is established in hot and dry environments [20,22,44]. Our results clearly indicate that the population of *T*. *granarium* increases with temperature up until 34.52°C. This is an important finding suggesting that global warming favors the increase of the population of this species. International trade in conjunction with global climatic change favors the dispersal of invasive species, like *T*. *granarium* [47]. Therefore, locations that are free of *T*. *granarium* but exhibit variable climatic conditions, compatible with those where *T*. *granarium* is already present, established or even intercepted should be on alert for the potential arrival of *T*. *granarium*. For example, the USA Government pays particular attention on phytosanitary measures and application of insecticides which aim to control *T*. *granarium* at the entry points of the country that are related to international trade [23,52,53]. The fact that about 84% of *Trogoderma* spp. intercepted at the US ports between 1985 and 2010 were *T*. *granarium*, while after 2010 *T*. *granarium* interceptions have been dramatically increased in the USA and several countries of Northern and Southern Europe, reveals the potential risk of further and rapid expansion of this species worldwide [23,54].

The reproductive value of females, that is the contribution an individual of a particular age will make to future generations [19], increases until a specific age. This is due to the early mortality of the pre-reproductive age classes of *T*. *granarium* and the subsequent increase of the age-specific fecundity. Thereafter, a decrease to the age-specific fecundity has a negative effect on the reproductive value which declines to zero for the older ages. Individuals of roughly 63, 42 and 21 days-old at 30, 35 and 40°C respectively reach their maximum reproductive potential. The expected remaining lifetime decreases until a specific age at 35 and 40°C due to early mortality, thereafter increases due to decreasing mortality, followed by an ultimate decrease. On the other hand, the expected remaining lifetime at 30°C is characterized by a continuous decrease due to no remarkable early mortality.

The process of *T*. *granarium* laying eggs was modeled by a Zero Inflated Poisson model [55]. Statistical learning for models of this kind represents a non-standard problem due to irregularities in the likelihood function and adopting a sampling-based approach to inference such as MCMC [31,56] offers a substantial advantage, including the ability to estimate the complete posterior distribution of the Poisson rate and the probability of excess zeros. The separation of this probability at 40°C compared to the other two temperatures is immediately apparent by simple visual inspection and this is represents a desirable feature of the proposed statistical analysis.

The stochastic approach to demography offers a number of additional advantages. Here we present an effort towards the parametric characterisation of the different durations [57] which represent the distinct components of the underlying biological process and in future research we shall endeavour to study the universality of these distributions by examining the parametric forms of these durations for related species. In addition, exploring the distribution of the time to the first birth naturally gives rise to an independent censoring mechanism necessitating a survival type of analysis for this component of our data.

Investigating for influential individuals is of paramount importance for robust statistical results. Such considerations are relatively straightforward when adopting a stochastic approach to demography and this aspect was examined in the present study by leveraging upon the posterior density [56], suggesting that the model appears to accommodate all the individual data reasonably well since no major departure from the bulk of the observations was observed.

In summary, the use of a deterministic approach of *T*. *granarium* growth provides estimates of its reproductive potential, an issue that should be taken in account in the study of its biology and be considered as an important component in the design of pest's management strategies. Furthermore, our approach could be considered as an additional tool in a broader sense, combined with models related to international trade and climatic change, since these models alert specialists towards early detection strategies against invasive species and consequently their successful control [47,58–61]. In addition, stochastic modeling of the variables (characteristics) of interest for *T*. *granarium* like their survival time, their time until first egg emerges or the number of eggs lying, provides an assessment of the variability for such variables, thus offering plausible ranges for use in alternative conditions (e.g. temperature, relative humidity, commodity), for comparison with different but related species. Also, the stochastic models of this study allowed for checking model fit and the characterization of the most suitable distribution for each component of the system, allowing respectively for robust results and casting the durations involved in this particular species within a wider taxa.

## Supporting information

S1 File Parametric models for survival data and Akaike Information Criterion. (DOCX)
