# Supplementary material for: Elucidating fitness components of the invasive dermestid beetle Trogoderma granarium combining deterministic and stochastic demography
Source: PLoS One. 2019 Feb 14;14(2):e0212182. doi: 10.1371/journal.pone.0212182 (PMC6375610; doi:10.1371/journal.pone.0212182)
Supplement: S1 File — (DOCX) [file pone.0212182.s001.docx]

Parametric models for survival data

The parametric model used to fit the *T. granarium* data is the accelerated failure-time (AFT) model which is a parametric model of the form: $S\left(t|\theta\right)=e^{X΄\theta}\cdot S_{0}\left(e^{X΄\theta}t\right)$;

where *S_0_* is a function for the baseline survival rate (which is related to the mean survival time). The AFT model uses covariates in term *e^X΄θ^* to place individuals on diﬀerent time scales. The term *e^X΄θ^* is called the acceleration factor as it scales (accelerates) the survival time for each covariate. The AFT model can be rewritten in a log-linear form, that is: $\log T=\mu+X΄\theta+\sigma W$;

where the logarithm of survival time *logT* is linearly related to its mean µ, to the acceleration factor *X΄θ*, and to an error term *σW* scaled by a parameter *σ*. Variable *W* describes the error distribution and is chosen among four well known distributions whose probability density functions are given below:

1. the **Exponential** with rate parameter *λ* (one degree of freedom)

$$p\left(w\right)=\lambda e^{-\lambda w}$$

1. the **Weibull** with shape parameter a and scale parameter b (two degrees of freedom)

$$p\left(w\right)=\frac{a}{b}\left(\frac{w}{b}\right)^{a-1}e^{-\left(\frac{w}{b}\right)^{a}}$$

1. the **Lognormal** with mean μ and st. deviation σ of the log(w) (two degrees of freedom)

$$p\left(w\right)=\frac{1}{\sigma w\sqrt{2\pi}}e^{\frac{-\left(\log w-\mu\right)^{2}}{2\sigma^{2}}}$$

1. the **Log-logistic** with shape parameter a and scale parameter b (two degrees of freedom)

$$p\left(w\right)=\frac{\frac{a}{b}\left(\frac{w}{b}\right)^{a-1}}{\left(1+\left(\frac{w}{b}\right)^{a}\right)^{2}}$$

In order to fit the AFT model to the *T. granarium* data, the survreg() function from the survival package in R is used.

Akaike Information Criterion

The Akaike Information Criterion (AIC) is an estimator of the relative quality of statistical models for a given set of data. It is defined as:

$AIC=-2\log\left(\hat{L}\right)+2k$;

where $\log\left(\hat{L}\right)$is the logarithm of the likelihood of the data given the current model evaluated at the maximum likelihood estimates of the parameters and *k* is the number of estimated parameters. AIC is a measure of the goodness of fit of a given model accounting for model complexity. So AIC is derived for each model by either calculating the
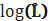
 and *k* manually using the results of the “survreg” function or by using directly the “extractAIC” function in the survival package in R.
